# A Model to Facilitate Resilience Among Diagnostic Radiography Students

**DOI:** 10.1002/jmrs.70107

**Published:** 2026-07-02

**Authors:** Heidi Thomas, Kathleen Naidoo, Penelope Engel‐Hills

**Affiliations:** ^1^ Cape Peninsula University of Technology Cape Town South Africa

## Abstract

**Introduction:**

A significant increase in the demand for radiography examinations and the corresponding rise in the workload of radiographers has been noted. Radiography imaging departments serve patients presenting with a wide range of conditions and abilities, and student radiographers have reported feeling unprepared, lacking confidence and experiencing stress and anxiety during such interactions. Additionally, they encounter challenges with effective communication and interpersonal engagement, making it difficult for them to recover from difficult experiences. These challenges highlight the need for research on resilience among student radiographers. The aim of the paper is to describe the process undertaken to develop a model to facilitate resilience in DR students, and to present the developed model.

**Method:**

A qualitative, exploratory, descriptive, contextual and theory‐generative approach was used to develop a model for facilitating resilience among diagnostic radiography (DR) students. Data were collected through focus groups with first‐year DR students. Finalisation of the model was informed through input by experts in model development, radiography and nursing education.

**Results:**

Five focus groups were analysed using thematic analysis, yielding four themes: students' understanding of resilience, students' readiness to commence in the clinical workplace, interpersonal interactions as they relate to the clinical environment and adapting to the clinical environment. Through inductive reasoning, central concepts were identified with facilitation, self‐efficacy and social connections, forming the basis of the resilience model.

**Conclusion:**

The model designed for the facilitation of resilience is recommended for use as a framework for educators to foster resilience among DR students.

## Graphical abstract

The model provides a framework for fostering resilience among diagnostic radiography students by strengthening self‐efficacy and establishing social connections.

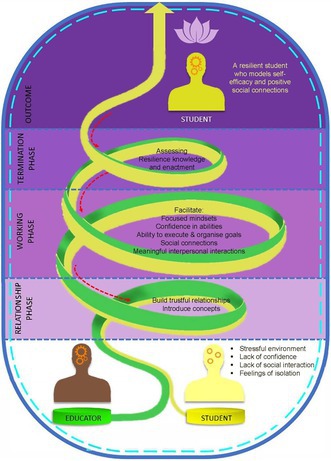

## 1 Introduction

Radiography education in South Africa requires students to complete both an academic programme and compulsory workplace learning (WPL) [1]. While the academic component provides theoretical knowledge, WPL immerses students in authentic clinical environments where they gain practical experience and develop professional competencies [2]. This work‐integrated learning (WIL) curriculum structure is designed to enhance students' workplace readiness and facilitate a seamless transition from the academic setting to professional practice.

However, the radiography profession is widely recognised as demanding and fast‐paced, characterised by high workloads, fast patient turnover and the need to make complex decisions involving imaging techniques for critically ill or injured patients [3, 4, 5]. Together with these pressures, radiographers are expected to place patients at the centre of care [6]. Patient interactions within radiography are diverse and often emotionally challenging, as patients may range from ambulant to post‐operative or in severe cases, comatose or near‐death [4, 7]. In addition to these encounters, students must navigate professional interactions with peers, radiographers and educators [8]. The expectation of being technically competent and compassionate towards patients and the need to navigate interpersonal interactions can be overwhelming [4, 8].

There has been a focus on the holistic development of students in healthcare professions to prioritise their well‐being [9, 10, 11]. Specifically, the concept of resilience has gained interest as it relates to the welfare of radiography students and has become a key graduate attribute [4, 11, 12, 13]. Resilience is referred to as ‘the ability of an individual to adjust to adversity, maintain equilibrium, retain some sense of control over their environment, and continue to move on in a positive manner’ [14].

Despite recognising resilience as important, there remains limited guidance on how this attribute can be effectively learnt and embedded within radiography curricula. Addressing this gap requires the development of practice‐based frameworks or models [15], that can guide educators to foster resilience among students. A model provides a visual or symbolic representation of a theory and serves as a practical tool for expressing abstract concepts in a clear and accessible manner [16]. In contrast, a theory is ‘an expression of knowledge within empiric patterns, the creative and rigorous structuring of ideas that project a tentative, purposeful and systematic view of phenomena’ [17]. Both are thus critical for enhancing clarity and accessibility, as well as for facilitating the understanding and explanation of a phenomenon.

### 1.1 Problem Statement

Many radiography students enter the clinical setting very early on in their training. Furthermore, radiography students have frequently reported experiencing uncertainty, a lack of confidence, nervousness, feeling overwhelmed and isolated [4, 18, 19, 20]. Despite the reported stressors, there is a scarcity of literature reporting on structured approaches that can be implemented by educators to facilitate resilience in radiography students.

### 1.2 Aim

The aim of the paper is to describe the process undertaken to develop a model to facilitate resilience in DR students, and to present the developed model. This addresses the overarching research question: How can resilience be developed in first‐year diagnostic radiography (DR) students?

## 2 Research Design and Method

### 2.1 Design

A qualitative, contextual and theory‐generative approach was adopted to guide the development of the model to facilitate resilience. The use of the theory‐generative approach was necessary to derive meaning from the findings and to put the relatively unexplored phenomenon of resilience into perspective by producing a concept to serve as a frame of reference for model development to enhance understanding and strengthen resilience in the radiography context.

### 2.2 Method

The model was generated following the steps of model development [21]. Steps 1 and 2, which involve concept analysis and the identification of relationship statements respectively, were informed by existing data collected over a three‐month period (December 2021–February 2022). The data were collected through five focus group interviews with a purposive sample of 21 first‐year diagnostic radiography (DR) students who volunteered and could provide comprehensive and pertinent insights into the topic.

After extensive engagement with the data, four themes, namely students' understanding of resilience, students' readiness to commence in the clinical workplace, interpersonal interactions as they relate to the clinical environment and adapting to the clinical environment, were constructed [22].

The primary focus was on using existing data from resilience research to develop a model. A limited number of verbatim quotations from the thematic analysis are presented below to support the use of the themes that enabled the identification of a central concept and relationship statements.

Theme 1: Students' understanding of resilience

Resilience to me is basically like, pushing through, like identifying your problem, or at least seeing that there is a problem, and then focusing on that problem. (FG1P2)

With resilience, it's just like overcoming that hardship. I think just speaking about it helps. (FG3P1)

Theme 2: Students' readiness to commence in the clinical workplace

Going to the hospital environment for the first time. I knew how to position a patient, but you know, there was no experience to it. So I was nervous. (FG1P2)

My first day, I was excited to be at the hospital, to experience what radiography is all about, and to know more about it. (FG2P3)

Theme 3: Interpersonal interactions as they relate to the clinical environment

Students should ask questions and not keep everything in … talking to someone could also solve the problem. (FG1P1)

There are staff who are actually really good in teaching, they give their time, they explain things thoroughly, and they just help us even with the reports that we ask them, they just do them willingly because they see we are trying by all means. (FG4P1)

Theme 4: Adapting to the clinical environment

Clinical is overwhelming, and it's scary at the same time … but they do give you room to make mistakes, and they also give you time to adapt in the environment and also learn how to do your patient and also learn your own ways on how to deal with patients. (FG5P3)

It feels like they don't understand what you are going through in the clinical. Clinicals are very draining. (FG4P1)

Using inductive reasoning, the researcher reflected on the themes to uncover an underlying and shared meaning across the four themes, from which the central concepts: facilitation, self‐efficacy and social connections were identified. These concepts were further validated through the researcher's field and reflective notes, compiled during data collection. Each concept was then defined within the study context, drawing on dictionary definitions and literature from health and social sciences. The defining attributes of each concept were subsequently identified, allowing for the construction of critical defining attributes. This process informed the formulation of a definition for the facilitation of self‐efficacy and social connections in DR students.

Step 2 involved developing relationship statements. These are statements that structurally connect the concepts of a theory, illustrating how they relate to and interact with one another in a way that is specific to the study context [21].

In Step 3, a model wherein resilience is emphasised to prepare first‐year DR students for the clinical environment was developed. The model was evaluated by experts in model development, radiography and nursing education, and their suggestions were incorporated before finalising the model.

### 2.3 Ethical Considerations

Ethical clearance (REC 2021/H27) was obtained from the Research Ethics Committee (REC) of the Faculty of Health and Wellness Sciences prior to commencing the study. DePoy and Gitlin [23] assert that participants of research studies must be informed of the purpose of the study and their agreement in terms of confidentiality as well as voluntary participation. In line with this, voluntary participation was sought from participants and their rights to privacy and confidentiality were protected. During the data collection stage, each participant was assigned a unique code concealing their identity. Participants could decline to participate in the study or decide not to continue at any stage without the risk of harm or consequences.

### 2.4 Trustworthiness

The rigour of the qualitative study was upheld by following trustworthiness principles. Guba and Lincoln's model describe the criteria for trustworthiness through four elements, namely credibility, transferability, dependability and confirmability [24]. Credibility refers to the extent to which the study accurately captures the phenomenon it intends to investigate. The researcher's awareness of qualitative research principles facilitated the truthful representation of findings. Established qualitative methods were employed, and a detailed research journal was maintained to document all phases of the research process. This journal included reflective notes and field observations, enhancing transparency and coherence. According to Holloway and Galvin [25], reflective documentation conducted alongside data collection and analysis contributes to triangulation, which strengthens credibility. An additional strategy to ensure credibility involved member checking, wherein the identified themes and categories were presented to participants for verification. This was done to eliminate potential misunderstandings and to ensure that participants' views and explanations were accurately captured [25, 26]. Transferability refers to the extent to which the research results may be applicable to other contexts [27]. To demonstrate the extent to which the results are transferable, an explanation of study context, the methods used to collect data and the sampling method is available. Shenton [27] explains dependability as the reliability and consistency of the findings and how much the study yields the same results should it be repeated in the same context. Dependability was ensured by following a systematic approach, documenting all steps in the research process and keeping an audit trail of focus group recordings, transcripts and field notes. This documentation confirms that the findings are grounded in the data and not influenced by researcher bias [27].

## 3 Results

Model development involved 3 steps: concept analysis (step 1), relationship statements (step 2) and model development (step 3) [21].

### 3.1 Step 1: Concept Analysis

Concept analysis is the first step in model development [21]. The concept analysis process outlined by Walker and Avant [28] was followed, which necessitated two phases, namely, selecting the concepts and defining and classifying concepts.

#### 3.1.1 Step 1 Phase 1—Selecting a Central Concept

In this study, the central concept was identified by exploring the influences of resilience together with how students defined resilience as it relates to the clinical environment. This was achieved through prolonged engagement with DR students during focus groups. Four main themes were developed: students' understanding of resilience, students' readiness for workplace learning, interpersonal interactions as they relate to the clinical environment and factors affecting the transition to the clinical environment. The results indicated that students experienced considerable negative emotions during their initial clinical placement, which negatively impacted their confidence. Students felt ill‐prepared for the various interactions in the clinical environment, with findings regarding interactions with qualified radiographers and lecturers highlighting the need to foster supportive relationships with students. Using an inductive reasoning approach, the researchers identified the central concept as: the facilitation of self‐efficacy and social connections in diagnostic radiography students.

#### 3.1.2 Step 1 Phase 2—Defining and Classifying Concepts

Concepts form the basic building blocks of theory generation. Walker and Avant [29] assert that defining concepts within the study context is critical as concepts can be interpreted in multiple ways depending on the disciplinary lens. The three concepts were defined, and stemming from this analysis, the definition developed for the facilitation of self‐efficacy and social connections in diagnostic radiography is: ‘Offering help and support to DR students to develop a focused mindset so that they have the capabilities to be successful at organising and executing a planned action. It is a process of supporting DR students to recognise their value within a group by connecting with others and developing positive interpersonal relationships’ [22].

The classification of the central concepts (Table 1) was based on the framework of Dickoff et al. [30].

**TABLE 1:** Classification of the central concepts.

The classification of the concepts for the model is
The agent in this model is the radiography educator. The radiography educator provides a conducive learning environment and facilitates the process of developing resilience in students
The recipient is a first‐year DR student who experiences the DR clinical environment and who needs guidance to develop resilience
The context is a higher education institution (HEI) where the student completes the DR training. Training is undertaken in two key settings, namely the classroom (based at the university) and the diagnostic clinical environment
The participants of the study clearly articulated the need for resources to buffer against uncertainty, anxiety and feeling overwhelmed. They spoke about the determination to push through and the need for confidence and believing in their ability when things were tough. They identified that social relationships in and outside of the radiography context influenced their resilience. Support from their family, friends, peers and radiographers was frequently required as a means of coping during challenging times. A lack of meaningful interaction and feelings of isolation were further highlighted. The curriculum, however, falls short of prescribing methods to develop resilience in radiography students to buffer against stressors of the clinical environment
The dynamics refer to the internal and external motivation of both the agent and the recipient to meet the outcome. The participants in the study clearly articulated the need for resources to buffer against uncertainty, anxiety and feelings of overwhelm. The determination to push through and the need for confidence and belief in their ability when things were tough were highlighted. Furthermore, they identified that social relationships, both within and outside the radiography context, influenced their resilience. Support from their family, friends, peers and radiographers was frequently relied on as a means of coping during challenging times. A lack of meaningful interaction and feelings of isolation were further highlighted. The educator's internal motivation is closely linked to her belief that demonstrating empathy and compassion towards patients and students is essential, as these actions help mitigate stress and anxiety. With appropriate support systems in place, anxiety‐inducing emotions can be alleviated. Additionally, the curriculum falls short of prescribing methods to develop resilience in radiography students to buffer against stressors of the clinical environment
The procedure reflects the processes that the radiography lecturer follows to develop resilience in students. The educator displays keen enthusiasm when introducing methods to cultivate resilience. The educator encourages students to actively participate in the learning process. The students are the recipients of structured methods which aims to enable focused mindsets, belief in abilities and successful organisation and execution of goals. The students are guided to make positive social connections and develop interpersonal relationships with peers, radiographers, clinical supervisors and lecturers

### 3.2 Step 2: Relationship Statements

The classified concepts served as the basis for forming relationship statements and demonstrate how they relate and interact with each other within the study context [21]. The relationship statements for this model are demonstrated in Table 2.

**TABLE 2:** Relationship statements.

In the model, the goal of the educator is to cultivate resilience in radiography students through a structured process of offering support and guidance. This goal will be achieved through the systematic provision of assistance and support by creating an environment in the classroom that fosters the development of resilience among radiography students through the facilitation of self‐efficacy and establishing social connections
The curriculum does not noticeably address the facilitation of resilience, thus lacking the inclusion of effective coping methods for radiography students faced with challenges in the clinical environment
The educator plays a pivotal role in empowering the radiography student to enhance their self‐efficacy and social connections by supporting, guiding and introducing methods to improve confidence and self‐belief and promote communication and interpersonal relations essential for developing resilience in radiography students
The radiography student actively embraces and applies the methods fostering a focused mindset, belief in their abilities and successful organisation and execution of planned actions. Furthermore, the students actively adopt and participate in the methods to establish positive social connections and interpersonal relationships involving peers, radiographers, clinical supervisors and lecturers
The embracement of self‐efficacy and social connections enables the resilience needed to cope effectively with the demands of the clinical environment

### 3.3 Step 3: Description of the Model

#### 3.3.1 The Purpose of the Model

**FIGURE 1:**
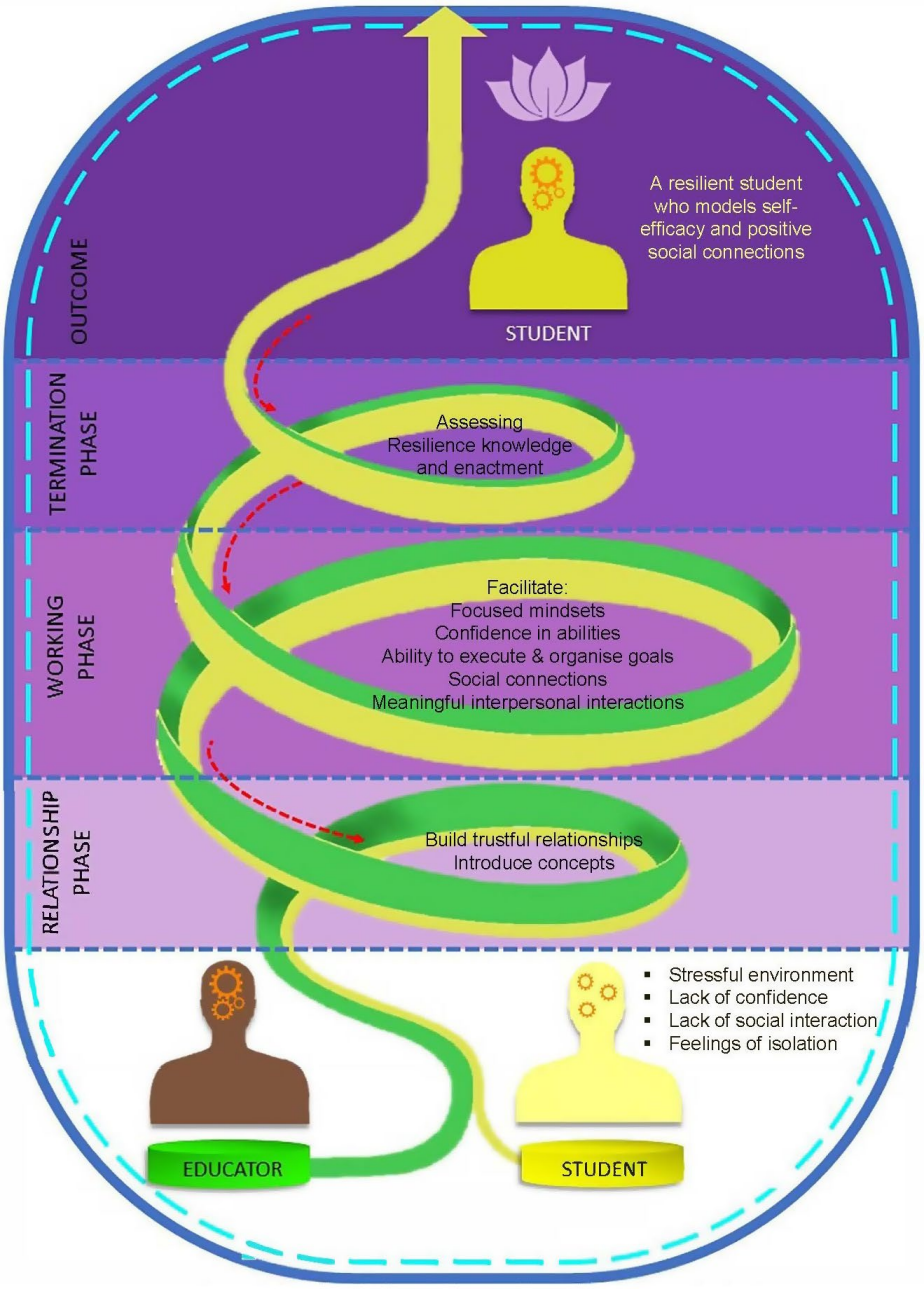
A model to facilitate resilience in radiography students.

The purpose of the model is to provide a framework for facilitating resilience among diagnostic radiography students. The model (Figure 1) is for use by educators training DR students.

#### 3.3.2 The Assumptions of the Model

The model is supported by three key assumptions. These assumptions are based on the meta‐theoretical foundation of the Health Promotions Model (HPM) [31]. The first assumption assumes that a person is autonomous and partially shaped by the environment thereby indicating that a person has the capacity to govern and make decisions for themselves without being coerced. The radiography student (person) is introduced to methods to cultivate resilience. The student, who is an autonomous being, voluntarily adopts and participates in these methods. The student's decisions are influenced by the internal and external environments; therefore, the person and environment cannot be seen in isolation but interdependent to the learning process. The second assumption of the HPM posits that a person thrives in an ‘environment’ where positive health‐enhancing behaviours are prioritised. By implication the internal environment, denoting cognitive and mental factors and the external environment, encompassing the physical and social context, must undergo transformation. By fostering self‐efficacy within the internal environment and encouraging social connections in the external environment, it is anticipated that a gradual and positive transformation towards the development of resilience will happen over time. Bandura [32] defines self‐efficacy as the individual's belief about their capability to complete a task successfully. These beliefs play a critical role in shaping how individuals view and respond to challenges within the clinical environment where students have reported a lack of confidence, fear of making mistakes, uncertainty and unpreparedness for diverse person interactions [4, 18, 33]. Furthermore, the literature explains self‐efficacy as critical for the development of resilience [34, 35, 36]. Like self‐efficacy, social connections are vital for fostering resilience. Research by Smith and Dhillon [37] reports that a lack of social connectedness resulted in radiography students feeling distressed, thereby compromising their resilience. Supporting this, research in education and psychology shows that social ties with family, friends and those in the workplace are critical for the development of resilience [38, 39, 40].

The third assumption of the theory emphasises the necessity of the ‘health profession’ to maintain favourable conditions contributing to healthy behaviours. Consequently, recognising that students' coping may be affected by the stressful and inherent complexities of the radiography clinical environment, efforts tailored to the demands of the profession are made to alleviate stressors. In this process, the educator adopts a crucial role in providing support and guidance to students by effecting the model in the classroom, fostering resilience that can be implemented in the clinical environment.

#### 3.3.3 The Context of the Model

The context is a radiography department/programme at a HEI. Within the research context, the radiography curriculum lacks explicit reference to resilience support methods.

#### 3.3.4 The Structure of the Model

- The model's context is visually indicated by two borders whereby the outer border refers to the classroom setting, and the inner border refers to the clinical environment where DR students gain real‐life workplace‐based experiences. Learning, interacting, communicating, growing and developing a focused mindset, and building positive interpersonal relationships happen in both these settings. However, methods to support the cultivation of resilience are facilitated in the classroom.
- The model starts with the radiography educator and the DR student coming together to commence the educational journey towards developing resilience as it relates to the clinical environment. Cogwheels are depicted in the heads of both the educator and the student, symbolising efficiency and productivity [41]. In the educator, interconnected cogwheels represent integrated knowledge, experience and the ability to apply understanding effectively within the learning environment. In contrast, the student's cogwheels are not yet interconnected, indicating foundational knowledge and emerging resilience, but a limited ability to integrate and apply learning. Through active engagement in the learning process, these connections develop, fostering cognitive growth and resilience.
- The educator's journey is denoted by the green platform and spiral, a deliberate choice due to the association of the colour green with growth, moving forward, reassurance and success [42]. On the other hand, the student's journey is indicated by the yellow platform and spiral. Yellow denotes enthusiasm, optimism and confidence [42, 43]. Radiography students often commence their educational journey possessing these attributes [4, 44]. The overarching goal is to harness and further cultivate these attributes as students embark on their educational journey; subsequently, extending into their professional careers.

#### 3.3.5 The Model Process

The model process is guided by the facilitative phases consisting of a relationship, working and termination phase [45]. For the resilience facilitation, an upward spiral was selected for its association with optimal functioning and enhanced social openness to exploratory activity [46]. The three loops with varying sizes mirror the degree of effort invested in each stage by the educator and student.

In the relationship phase the emphasis is on facilitating a positive and trusting relationship between the educator and the student within a supportive learning environment. Additionally, a foundational understanding of key concepts of self‐efficacy and social connections critical for the development of resilience is nurtured. The supportive learning environment is important for creating a sense of safety and openness where students can openly and honestly express their viewpoints. It reflects a place where students feel at ease seeking guidance and exploring knowledge, and where a welcoming and caring atmosphere is given preference [47, 48, 49]. Toerien et al. [45] emphasise the importance of foundational understanding of issues at hand; therefore, an understanding of resilience, self‐efficacy and social connections is prioritised early on in the learning process. When these conditions are met, students are more likely to experience an increased sense of self‐efficacy, feel encouraged to participate and engage in learning and consequently foster a greater belief in their ability to succeed [50].

The working phase involves equipping students with support structures to facilitate focused mindsets, confidence in abilities, skills to organise and execute planned actions and the establishment of social connections and positive interpersonal relations. This comprehensive approach is necessary, aiming to enable students with a growth mindset, thereby viewing their knowledge, understanding and skills as abilities that can be improved through effort, whereas those with a fixed mindset view these abilities as stable and unchangeable over time [51].

In the radiography setting it is critical to be adaptable where interacting with diverse patients; the fast pace and evolving technology is inevitable. A fixed mindset can be limiting in this regard. Drawing attention to positive coping skills like self‐motivation and focusing on the end goal is specifically useful in creating a growth mindset and resilience. While a growth mindset is essential for resilience development, effective teaching strategies remain vital [51, 52].

The next step within the working phase focusses on nurturing confidence in abilities. Literature demonstrates that students with confidence in their abilities are more likely to take chances, engage in conversations and demonstrate more resilient behaviour when faced with difficulty [34, 53, 54]. Consequently, it becomes imperative to emphasise that radiography students develop confidence in their own abilities to facilitate resilience.

Execution of and organisation of goals represents another critical component of the working phase. Students frequently report experiencing anxiety and fear when facing emotionally demanding clinical situations. Enhancing logical thinking processes requires continuous practice and refinement of skills. The demands of adapting to an unfamiliar, high‐pressure environment can significantly affect their ability to execute and organise goals effectively, undermining their confidence.

Also linked to the working phase, the educator focusses on prioritising the facilitation of social interactions and positive relationships. Consequently, active interaction and bonding with peers, radiographers, educators and family members is actively encouraged, with a particular emphasis on dedicating time for meaningful interactions and embracing one's role as a valued member of a team.

In the termination phase, the students are assessed to determine whether they have developed resilience skills, thereby effectively demonstrating self‐efficacy and the ability to establish and maintain positive social connections.

#### 3.3.6 The Outcome of the Model

The outcome of implementing the model is enhanced resilience for a student who effectively models self‐efficacy and social connections in the clinical environment. At the beginning of the journey, the student is depicted in a pale yellow, with some resilience built through their life experiences. This transitions to a bright, intense yellow as the outcome. Bright colours are associated with freshness, enlightenment and positivity [55]. Specifically, yellow symbolises optimism, enthusiasm, confidence, emotional strength and intellect, suggesting the student's positive approach to the clinical learning environment and their professional role. The inclusion of a lotus flower at the model's outcome further reinforces this development, as it symbolises resilience and strength [56].

## 4 The Evaluation of the Model

The model was evaluated for its adequacy and applicability to practice using the criteria of clarity, simplicity, generality, accessibility and importance [21]. The evaluation panel consisted of experts with extensive experience within the professional fields of nursing and radiography and experience in model development ranging from one to more than seven years. All evaluators were independent from the study.

### 4.1 Clarity of the Model

The evaluators confirmed that the principle of clarity was met, as the concepts were presented clearly and in an understandable manner. To further enhance clarity, the previously solid phase lines were changed to dashed lines to indicate fluidity between phases and the figures within the model were labelled.

### 4.2 Simplicity of the Model

Evaluators agreed that the model achieved the standard of simplicity, as relationships were articulated in clear, simple statements, making it accessible and easy to apply.

### 4.3 Generality of the Model

Although the theory was developed for the context of radiography, it met the principle of generality, thereby suggesting its relevance and applicability to other situations. The evaluators affirmed that the model successfully met the generality principle, noting its potential transferability.

### 4.4 Accessibility of the Model

The evaluators confirmed that the model demonstrated accessibility, as the concepts were readily understandable and could be readily translated into practice. Furthermore, they emphasised the need to disseminate the findings so that those with an interest in the model can access the knowledge it conveys.

### 4.5 The Importance of the Model

An important theory is forward‐looking, usable across practice, education and research and is valued for guiding a desired outcome [21]. Evaluators considered the model both meaningful and valuable for supporting resilience building in the education context.

## 5 Limitations

Although the model was evaluated to be suitable and transferable to similar health‐related contexts where students commence workplace learning early in their training, the practical effectiveness of the model has not yet been assessed.

## 6 Conclusion

The study highlights a range of challenges experienced by DR students, including fear of the unknown, uncertainty, lack of confidence, isolation and challenges in communication during workplace learning. Higher education institutions play a critical role in addressing these concerns by integrating resilience methods into the radiography curriculum and equipping students to effectively navigate challenges inherent to the clinical environment. It is recommended that radiography educators foster a supportive and inclusive learning environment, incorporating both discussions and foundational teaching on resilience. A particular emphasis on strengthening self‐efficacy and fostering social connections should be prioritised, as they are critical components of cultivating resilience and supporting students' holistic development.

## Ethics Statement

Ethical clearance (REC 2021/H27) was obtained from the Research Ethics Committee (REC) of the Faculty of Health and Wellness Sciences, Cape Peninsula University of Technology (CPUT) prior to commencing the study.

## Conflicts of Interest

The authors declare no conflicts of interest.

## Data Availability

The data that support the findings of this study are available on request from the corresponding author. The data are not publicly available due to privacy or ethical restrictions.
